# An updated investigation on the dromedary camel cerebellum *(Camelus dromedarius)* with special insight into the distribution of calcium-binding proteins

**DOI:** 10.1038/s41598-020-78192-7

**Published:** 2020-12-03

**Authors:** Abdelraheim H. Attaai, Ahmed E. Noreldin, Fatma M. Abdel-maksoud, Manal T. Hussein

**Affiliations:** 1 Department of Anatomy and Histology, Faculty of Veterinary Medicine, Assiut University, 71526 Assiut, Egypt; 2 Department of Histology and Cytology, Faculty of Veterinary Medicine, Damanhour University, 22511 Damanhour, Egypt

**Keywords:** Cell biology, Neuroscience, Anatomy

## Abstract

Studying the cerebella of different animals is important to expand the knowledge about the cerebellum. Studying the camel cerebellum was neglected even though the recent research in the middle east and Asia. Therefore, the present study was designed to achieve a detailed description of the morphology and the cellular organization of the camel cerebellum. Because of the high importance of the calcium ions as a necessary moderator the current work also aimed to investigate the distribution of calcium binding proteins (CaBP) such as calbindin D-28K (CB), parvalbumin (PV) and calretinin (CR) in different cerebellar cells including the non-traditional neurons. The architecture of camel cerebellum, as different mammals, consists of the medulla and three layered-cortex. According to our observation the cells in the granular layer were not crowded and many spaces were observed. CB expression was the highest by Purkinje cells including their dendritic arborization. In addition to its expression by the inhibitory interneurons (basket, stellate and Golgi neurons), it is also expressed by the excitatory granule cells. PV was expressed by Purkinje cells, including their primary arborization, and by the molecular layer cells. CR immunoreactivity (-ir) was obvious in almost all cell layers with varying degrees, however a weak or any expression by the Purkinje cells. The molecular layer cells and the Golgi and the non traditional large neurons of the granular layer showed the strongest CR-ir. Granule neurons showed moderate immunoreactivity for CB and CR. In conclusion, the results of the current study achieved a complete map for the neurochemical organization of CaBP expression and distribution by different cells in the camel cerebellum.

## Introduction

The cerebellum plays a fundamental role for diverse motor functions, postural control, balance, and motor coordination^1,2^. It is also involved in cognitive functions such as language, learning, memory, and some emotional behaviors such as fear^3,4^. The cerebellum consists of 2 main components, the cerebellar cortex and the medulla. There are several cellular components in the cerebellar cortex; the most distinguished morphologically are Purkinje cells (PCs), granule, Golgi and basket cells^5^. This complex neural network gives rise to a massive signal-processing capability that plays a role in motor learning^6^. The inputs to the cerebellum come from the climbing fibers and the mossy fibers. Most of the neuronal subtypes in the cerebellar cortex are interneurons which synapse with PCs. The later's axons constitute the outputs, of the integrated signals, which rely on either small deep cerebellar nuclei lying in the interior of the cerebellum or transmit information to structures outside the cerebellum.

Calcium ions (Ca^+2^) are an important moderators, acting as major secondary messengers, of several vital physiological processes, including differentiation and morphogenesis, membrane excitability and conductivity, axonal transport, synthesis and release of neuroactive substances and some neurotransmitters (phenomena of synaptic plasticity). The calcium signal is mediated by a superfamily of structurally related proteins, generally known as calcium-binding proteins (CaBP), such as CB, PV, CR and S100 protein^7–11^. These molecules possess characteristic structures which can bind Ca^+2^ with high affinity^12–14^. Altered CaBP expression may lead to pathological and neurodegenerative conditions, such as in patients suffering from epileptic seizures are lacking neuronal PV^15–17^. It is important to study the cerebella of different animals, in order to expand our knowledge about the cerebellum and many cerebella of different species have been studied. However, there is a paucity of information concerning the morphology of camel cerebellum, even in the Middle East and Asia, where camels are important in their economies. It will be interesting to explore the cerebellum of an animal such as the dromedary, with very long legs and neck, who lives in a hot desert and walks long distances under hard conditions. All these factors may require specialized architecture in its cerebellum. Therefore, the present study was designed to achieve a detailed description of the morphology and cellular organization of the camel cerebellum. Because of the high importance of the calcium ions as a necessary moderator the current work also aimed to investigate the distribution of calcium binding proteins (CaBP) such as calbindin D-28K (CB), parvalbumin (PV) and calretinin (CR) in different cerebellar cells including the non-traditional neurons.

## Material and methods

### Collection of specimens

The Ethics Committee of Alexandria University, Egypt approved this study. In this study camel's brain were collected from ten clinically healthy mature camels (Camelus dromedarius) (4–6 years old) heads without sex differentiation. The animals were sacrificed in Alexandria slaughterhouse in Alexandria province, Egypt according to local ethical board guidelines of Egyptian slaughterhouses (Alexandria animal ethics committee 24122018). After slaughtering the camel, the entire head was separated from the neck, and saline was injected in the common carotid artery to wash the blood from the head circulation. The 4% paraformaldehyde in PBS was injected in the common carotid artery for at least 30 min then the skull was opened to obtain the brain. Every brain was hemisected into two halves before immersed in the same fixative to allow more exposure to the fixative. After have been stored at 4° C for 3 days. The cerebellum was carefully dissected from the brain and the weight of the brain and the cerebellum were weighed using sensitive balance.

### Light microscopic study

The tissue blocks were prepared for paraffin cutting. Briefly, the dehydration of the fixed tissues was carried out using ascending grades of ethyl alcohol. The samples were cleared by immersing in xylene then embedding in paraffin wax (Sigma Aldrich, USA) according to the standard methodology described by Romeis^18^. Step serial sagittal Sects. (100 serial sections) from the vermal region of folia VI, VII, VIII were cut at 6–8 µm thickness using a Richert Leica RM 2125 Microtome, Germany, and mounted on glass slides. Some sections were stained with cresyl violet (to stain cell bodies) together with luxol fast blue (for myelinated fibers) and other section with Grimelius silver nitrate impregnation (for both cell bodies and processes)^19^.

### Immunohistochemical staining

Immunohistochemical staining had been performed on the paraffin blocks as mentioned before by Abdel-Maksoud, et al.^20^. Sections were deparaffinized with xylene and hydrated with a descending grade of ethanol then washed with 0.1 M PBS (3 × 10 min). To decrease the masking of antigen epitopes, the antigen retrieval was carried out using 0.1 M sodium citrate buffer solution (pH = 6) for 7 min using a microwave (600 W). Then, sections were cooled to room temperature for 20 min and washed with PBS (pH 7.4) for 10 min. After blocking the endogenous peroxidase activity with 3% H_2_O_2_ in H_2_O for 30 min at the room temperature (RT), the sections were washed with PBS (3 × 5 min), then the sections were blocked with 10% normal donkey serum (NDS) + 0.2% Triton-X100/PBS for 2 h at RT. Subsequently, sections were incubated overnight at 4 °C with the following antibodies: rabbit polyclonal anti-CB [1:200, Genemed Biotechnologies, catalogue NO. (61-0061)], rabbit polyclonal anti-PV [1:200, Thermo Fisher Scientific catalogue NO. (PA1-3945)] and mouse monoclonal anti-CR [1:200, Thermo Fisher Scientific; catalogue NO. (MA1-16629)]. Sections were rinsed 3 × 10 min in 0.2% Triton-X 100/PBS and incubated with biotinylated IgG goat anti-rabbit (catalogue NO.E043201-8) and IgG goat anti-mouse secondary antibody (catalogue NO.P0447) (from Dako, Hamburg, Germany) diluted at 1:200 for 2 h at RT, followed by incubation with Vectastain ABC (Avidin–Biotin complex) reagent for 45 min in a humid chamber at room temperature. Visualization of the reaction was carried out with 0.04% 3,3′-diaminobenzidine (DAB) and 0.003% H2O2 in 0.05 M Tris–HCl buffer (pH 7.5) for 5–10 min. The sections were dehydrated in a graded series of ethanol, cleared with xylene and covered with DPX. Negative control were performed by incubating sections without the primary antibodies. Immunohistochemical staining was evaluated by LeitzDialux 20 Microscope and photos were photographed by canon digital camera (Canon Powershot A95) in the department of anatomy and histology, faculty of veterinary medicine, Assiut University, Egypt.

### Morphometric and statistical analysis

The morphometric studies were performed on both light and immunohistochemical images of the camel cerebellum using Image-J software. The dimensions and counting of the cells were performed by measuring at least 10 sections from every cerebellum, and the results were presented as the mean of the measurements and considered as representative at these regions. The cross sectional area (CSA) is the measured area of the cells containing the whole view of the nucleus. The diameters are the longest and shortest diameter of the cells, containing the complete view of the nucleus, passing through the nucleus. The cellular density per CSA was performed by counting cells in a defined area and were finally calculated to 0.01 mm^2^. The linear density for PC was performed by counting the PC in a defined length of 1 mm. We chose the linear density for PC because they appear in the sections arranged in a line between the granular and molecular layer. To evaluate the ratio of immunopositive PC for different markers, we counted 100 PC manually from 3 different sections per animal. Some measurements for the size and density of PC and granular cells are summarized in Table 1 .

**Table 1:** The dimensions and densities of the granule cells and Purkinje cells in the camel cerebellum. (CSA); cross sectional area.

CSA (μm2)	Diameter (μm)	Density (/0.001 mm^3^)	Density per CSA (0.01 mm2^)^
**Granule cells**			
35.7 ± 2	7.1 × 6.2	1289 ± 148	80.6 ± 9.26

CSA (μm2)	Diameter (μm)	Density (/0.001 mm^3^)	Linear density (/1 mm)
**Purkinje cells**			
797.73 ± 90	43.76 × 31	139.4 ± 12.8	7.4 ± 1.1

## Results

### Anatomical observation

**Figure 1:**
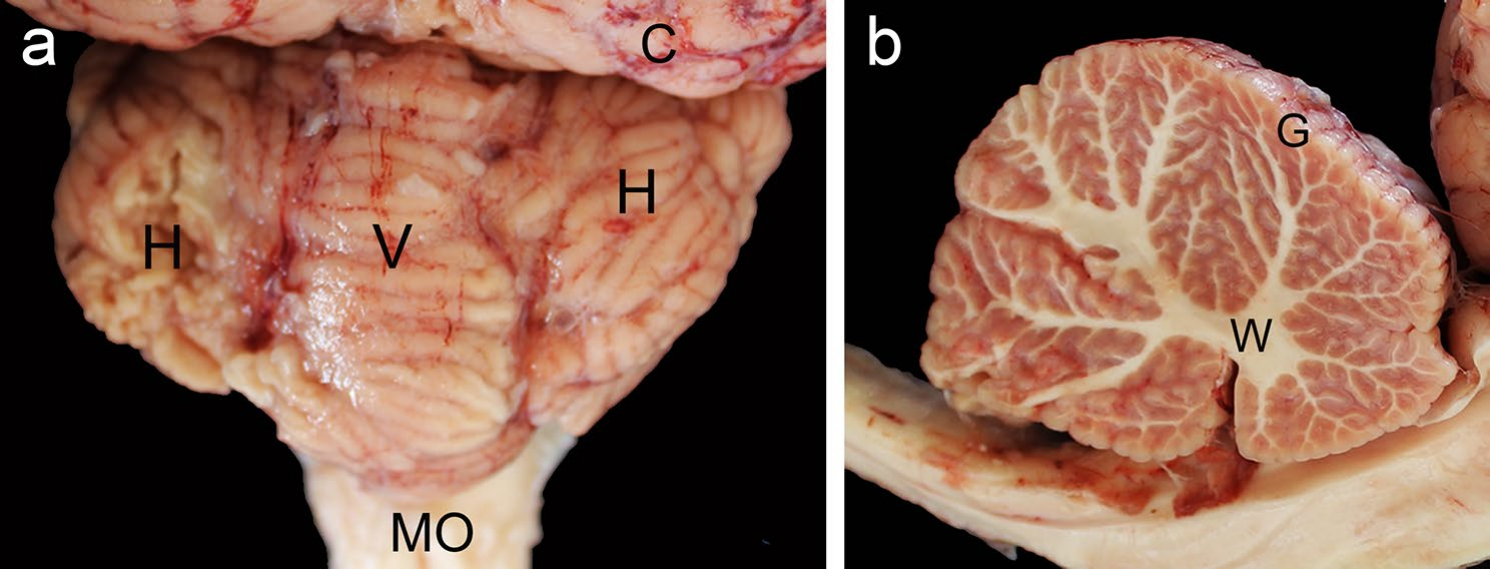
The anatomy of the camel cerebellum. (**a**) The camel cerebellum lies caudal to the cerebral hemisphere and above the medulla oblongata. It consists of a central vermis which flanked by the cerebellar hemispheres. Its surface shows a lot grooves. (**b**) A sagittal view of the camel cerebellum shows the characteristic central white matter which covered by the gray matter.

The camel cerebellum weighed 49.2 gm and the entire brain weighed 355 gm, therefore, the camel cerebellum averages 13.86% of the brain. The anatomical features of the camel cerebellum were comparable to those of other mammals. The cerebellum lies caudal to cerebral hemispheres. It consists of 2 symmetrical lateral halves, the 2 cerebellar hemispheres which flank the medial part, the vermis. The dorsal surface of camel cerebellum has many fissures of variable depths subdividing the entire cerebellar surface into a considerable number of leaf-like lamellae (cerebellar folia) which separated by sulci (of variable depth) (Fig. 1a). The internal branched white matter is covered by the foliated outer grey matter giving it the appearance of a tree, and hence its older name, the arbor vitae (Fig. 1b).

### The microanatomy

**Figure 2:**
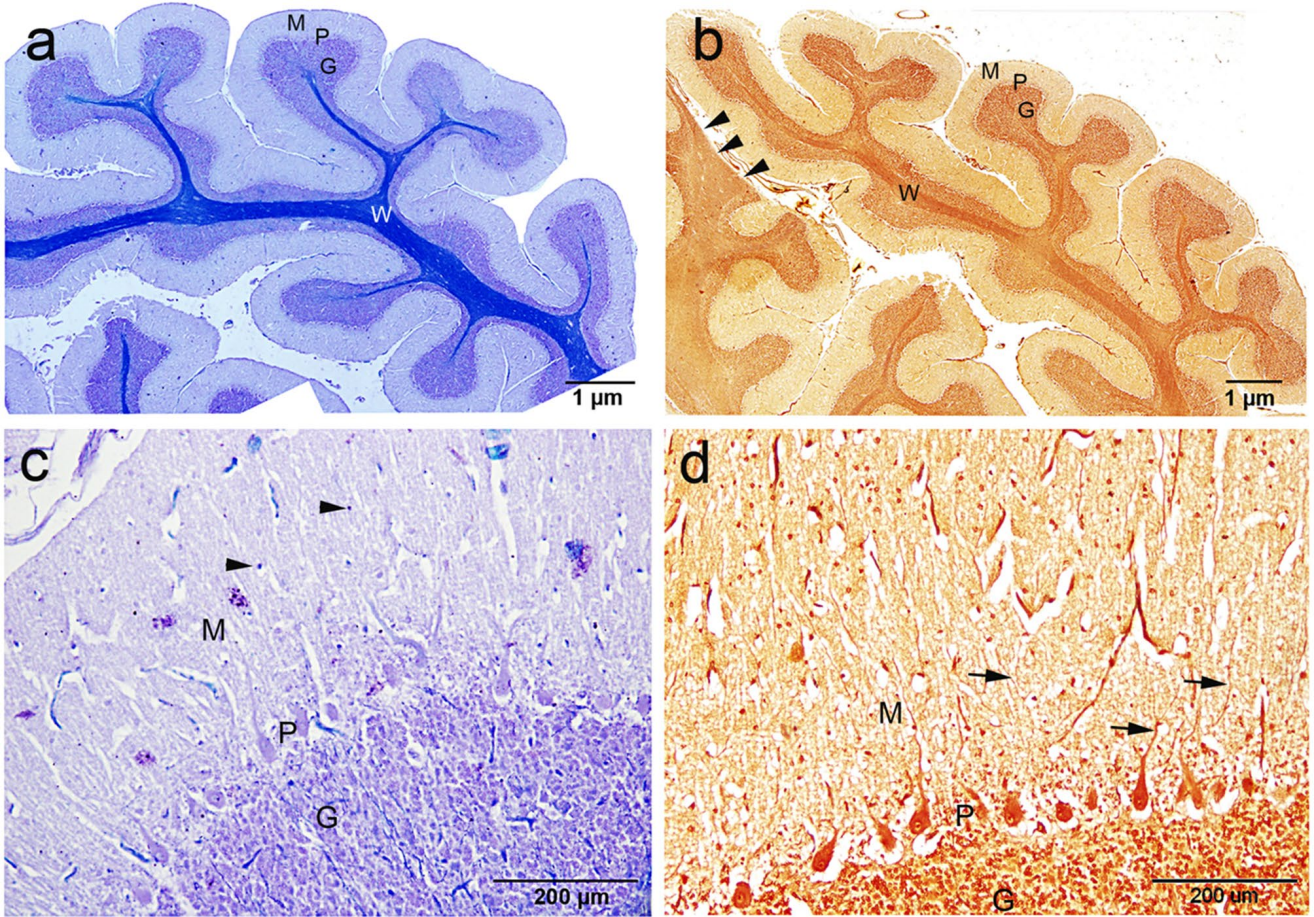
Histochemical staining showing the general architecture of the camel cerebellum. (**a**) Cresyl violet with luxol fast blue, (**b**) silver staining showing the cerebellar medulla white matter (W) and three layers of the cerebellar cortex; M (molecular layer), P (Purkinje layer) and G (Granular layer). (**b**) Notably, a wide region of white matter that reach distally to the pial surface, without cortical tissue covering (arrowheads). (**c**) The outer molecular layer (M) consisted of relatively few numbers of neuronal cell bodies (arrowheads). (**d**) Silver staining showing more polymorphic neuronal cells in the molecular layer compared to cersyl violet staining. Moreover, different processes of numerous cells could be visualized by the silver stain (arrows).

W applied some histochemical staining, such as cresyl violet (cell bodies) together with luxol fast blue (myelinated fibers) and silver impregnation (for both cell bodies and processes) on the camel cerebellar tissues to visualize the cytoarchitecture of the different types of neurons, dendrites and axons,. Histologically, the architecture of the camel cerebellum is conserved as different mammals and consists of the medulla covered by 3 layered-cortex; made up of an outer molecular layer, middle ganglionic (Purkinje) cell layer and inner granular layer (Fig. 2a,b). Surprisingly, we noticed a wide region of white matter that reach distally to the pial surface, without covering with cortical tissue (Fig. 2b, supplementary file).

**Figure 3:**
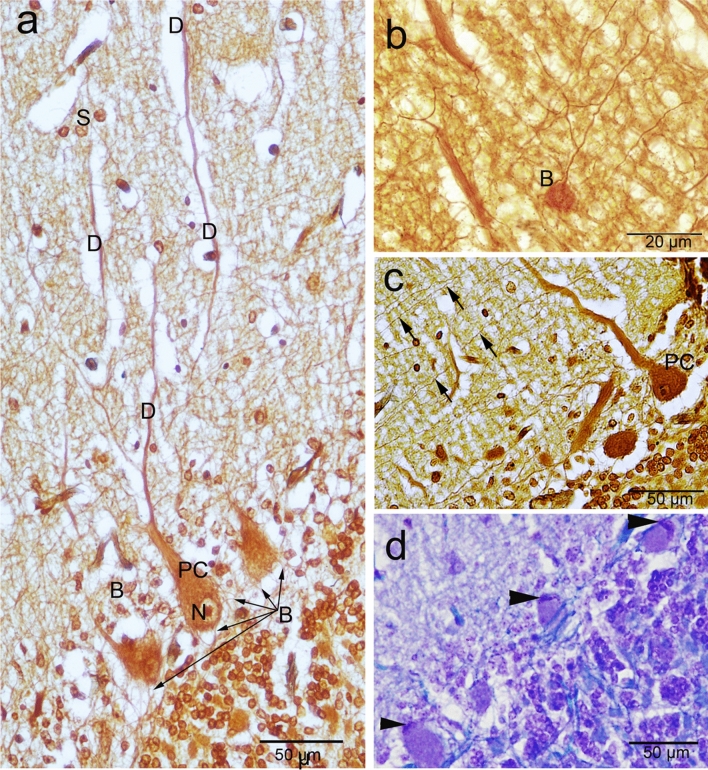
Histochemical staining showing the camel cerebellar cortex. (**a**) Silver staining showing the dendritic arborization (D) of the Purkinje cells which extend through the molecular layer. The outer half of the molecular layer contains stellate cells (S), and basket cells (B). The axons of basket cells make a meshwork around the cell bodies of Purkinje cells (arrows). The Purkinje cells (PC) in camel were characterized by their large, flask-shaped cell bodies. Each cell contained a vesicular nucleus (N) with a prominent circular nucleolus. (**b**) Basket cell dendrite (B) extends perpendicular to the dendretic tree of PC. (**c**) Silver staining showing the parallel fibers (arrows). (**d**) Cresyl violet staining showing the Nissl's granules (arrowheads) which arranged peripherally in the Purkinje cells at certain focuses.

The outer molecular layer consisted of relatively few neuronal cell bodies and many unmyelinated fibers (Fig. 2c). Polymorphic neuronal cells were observed in the molecular layer and the outer half showed more stellate cells, than the basket cells found in the deeper half, (Fig. 2d). Notably, the cresyl violet did not show as many cells in the molecular layer compared to the silver stain. Moreover, different processes of numerous cells could be visualized by the silver stain (Fig. 2c,d). The dendrites of the PCs appear to extend through the molecular layer (Fig. 3a). The axons of basket cells make a meshwork around the cell bodies of PCs (Fig. 3a), while their dendrites extend perpendicular to the dendretic tree of PC (Fig. 3b). The axons of granular cells traversing the molecular layer to variable depth then bifurcated at right angles to give rise to parallel fibers (Fig. 3c). Whereas, both silver and Luxol fast blue could not visualize the climbing fiber nor the mossy fibers.

The PC layer made up of one row of cell bodies was located at the border between the molecular and granular layers. The PCs in camel were characterized by their large, flask-shaped cell bodies. Each cell contained a vesicular nucleus with a prominent circular nucleolus (Fig. 3a,c) and we observed some Nissles granules arranged peripherally at certain focuses (Fig. 3d). Silver staining showed various processes synapsing with the cell bodies of the PCs. The PCs dimension measured 43.76 × 31 µm, with a CSA (cross sectional area) of 797.73 ± 90 µm^2^. PC linear density was 7.4 ± 1.1 cells/1 mm. The calculated density was 139.4 ± 12.8 cells/0.001 mm^3^ volume. Some of the measurements for the size and density of PC and granular cells, using ImagJ software, are summarized in Table 1 .

**Figure 4:**
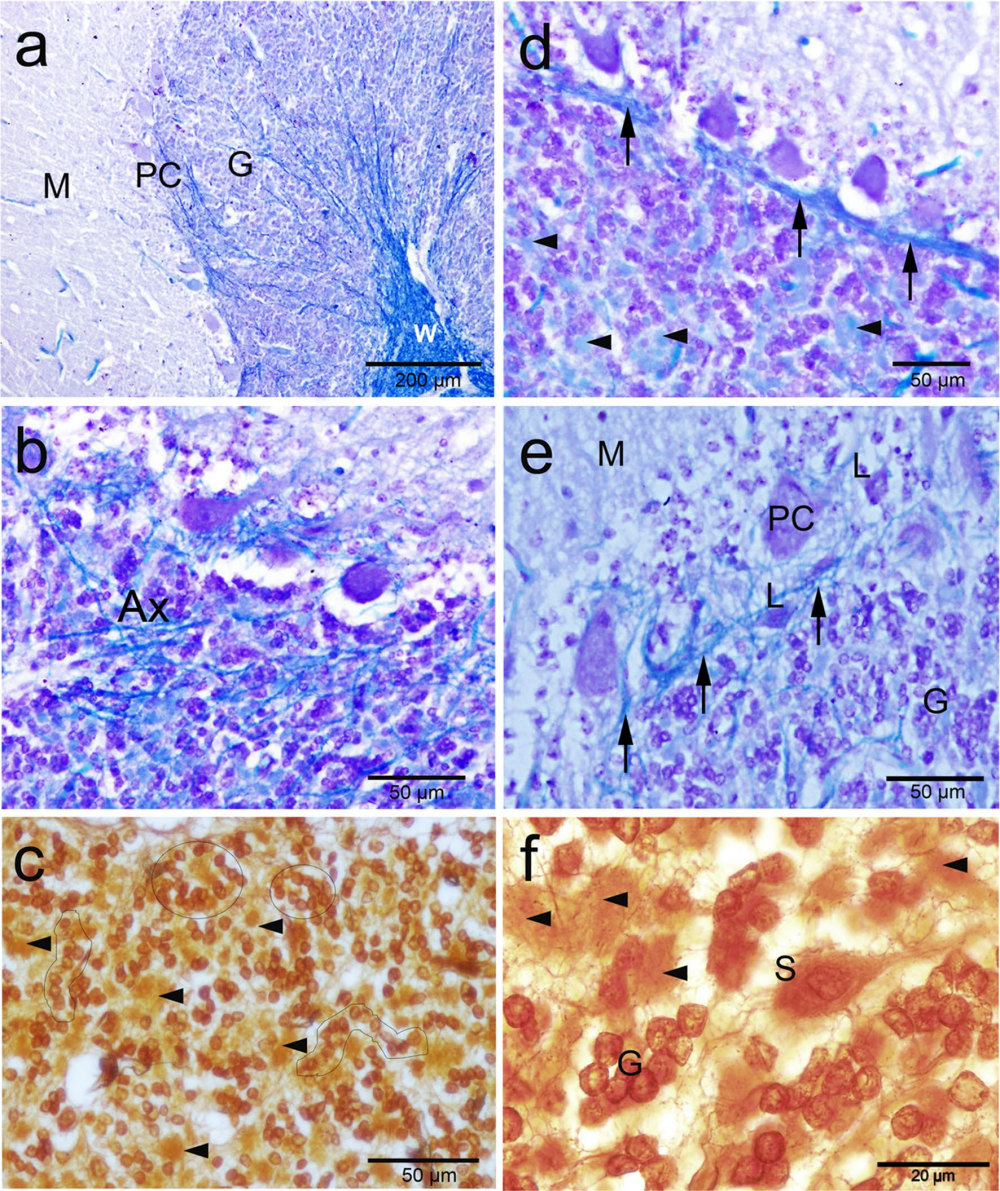
Histochemical staining showing the camel cerebellum. (**a**, **b**) Cresyl violet staining showing the granular layer cell layer which is extremely cellular and populated by small spheroid bodies. Luxol fast blue staining showing the white matter (W) and many myelinated axons (Ax) traverse through the granular either individually or in bundles. (**b**) Axons of basket cell surround the Purkinje cells (arrowheads). **c**: Silver staining showing the granular cells arranged in, rosettes (circles) or cords (irregular shapes). (**d**,** e**) Bundles run just beneath and parallel to the arranged Purkinje cell bodies (arrows). Arrowheads show the glomerular islands. (**e**,** f**) Non-traditional large neurons, such as Lugaro (L) and synaromatic neurons (S) with cresyl violet and silver stains respectively.

The inner most granular layer is extremely cellular and densely populated by small spheroid somata with dark-stained nuclei and scanty cytoplasm (Fig. 4a,b). The granular cells arranged in clumps, rosettes or cords, and many spaces exist within the granular layer, most of them are lightly stained with silver stain (Fig. 4b,c). The granule cell measured 7.1 × 6.2 µm with CSA 35.7 ± 2 µm^2^. Granule cell density was 80.6 ± 9.26 cells/0.01 mm^2^ CSA. The calculated density was 1289 ± 148 cells/0.001 mm^3^ volume.

Luxol fast blue staining showed that white matter and many myelinated axons traverse through the granular layer from the Purkinje cell layer and the molecular layer either individually or in bundles (Fig. 4a,b). Additionally, many luxol fast blue stained endings surround the somata of the PCs, which are likely from axons of basket cells (Fig. 4b). Occasionally, some bundles run immediately beneath and parallel to the arranged PC somata (Fig. 4d,e), which are likely axons from Lugaro cells, basket cells as well as collaterals from PC axons. Moreover, we were able to detect non-traditional large neurons, such as Lugaro (Fig. 4e) and synaromatic neurons (Fig. 4f), with cresyl violet and silver staining.

### Molecular characterization of the CaBP in camel cerebellum

Immunohistochemical staining against different molecules of the CaBP in camel cerebella such as CB, PV and CR were performed. However, immunopositive structures were detected throughout the cerebellar cortex, with layer-specific patterns of expression. The immunoreactivity was visualized in neuronal bodies, and in some instances, in dendrites and axons. Negative control sections incubated without the primary antibodies revealed no staining for neurons or fibers (See supplementary file).

### Calbindin-D 28 k (CB) expression in the camel cerebellum

**Figure 5:**
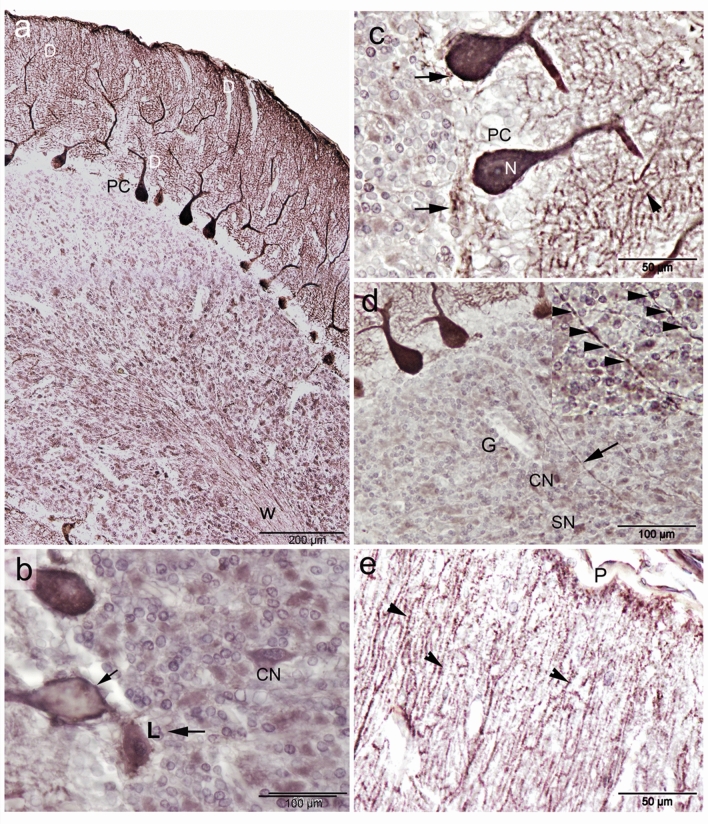
Calbindin-D28k (CB) immunoreactivity in camel cerebellum. (**a**) CB immunoreactivity was obvious in almost all cerebellar cortical layers and the white matter (W). Purkinje cells (PC) showed the highest CB immunoreactivity. In addition, the dendrites extend the whole length of the molecular layer, showing an obvious dendritic arborization (D). (**b**) Seldom low CB-immunopositive PC bodies were observed (arrow). Neuron of Lugaro (L) and candelabrum neurons (CN) were observed. (**c**) CB immunoreactivity was seen as densely packed homogeneous deposits in the cytoplasm of the Purkinje cells and within the nuclei (N). Notice, CB immunoreactive fibers beneath and surrounding the PC, which are likely from basket and/or Lugaro cells (arrow). Immunoreactivity was observed in the spiny branchlets (arrowhead). (**d**) CB-immunopositive fibers (arrow) were traversing through the granular layer (G) and were oriented vertically till reaching the white matter. Inset showing the varicosities (arrowheads) along the axon profile which originated from the PC. (**e**) CB immunoreactivity was observed along the dendritic arborization till reaching the pial surface (P), arrowheads indicates the spiny branchlets of the dendrities.

The Purkinje neurons showed the highest CB immunoreactivity, which demarcates the characteristic morphology of the Purkinje neurons throughout its extent (Fig. 5a). Moreover, the dendrites extend the whole length of the molecular layer and showing obvious dendritic arborization including the primary and secondary trunks and the spiny branchlets (Fig. 5a). However, seldom low CB-immunopositive PC bodies were observed (Fig. 5b). The ratio of CB-immunopositive cells were about 99 percent of the PC. The nature of the strong immunoreactivity was seen in densely packed homogeneous deposits in their cytoplasm, and in some cases, a similar immunoreactivity was seen within the nuclei (Fig. 5c,d). Strong CB-ir fibers were seen beneath and surrounding the PC, which are likely from basket and/or Lugaro cells (Fig. 5c). Also, some coarse CB-immunopositive fibers were traversing through the granular layer and were oriented obliquely or vertically till reaching the white matter. They are characterized by varicosities along with their profile and some of them could be traced originated from PC (Fig. 5d). Interestingly, the immunoreactivity was observed in the spiny branchlets with many irregularities on their surface indicating dendritic spines (Fig. 5e). There are also abundant immunopositive punctate elements (puncta) distributed in the neuropil between neurons of the molecular layer (Fig. 5e). All of the abovementioned immunopositive structures impart an intense coloration to the.

**Figure 6:**
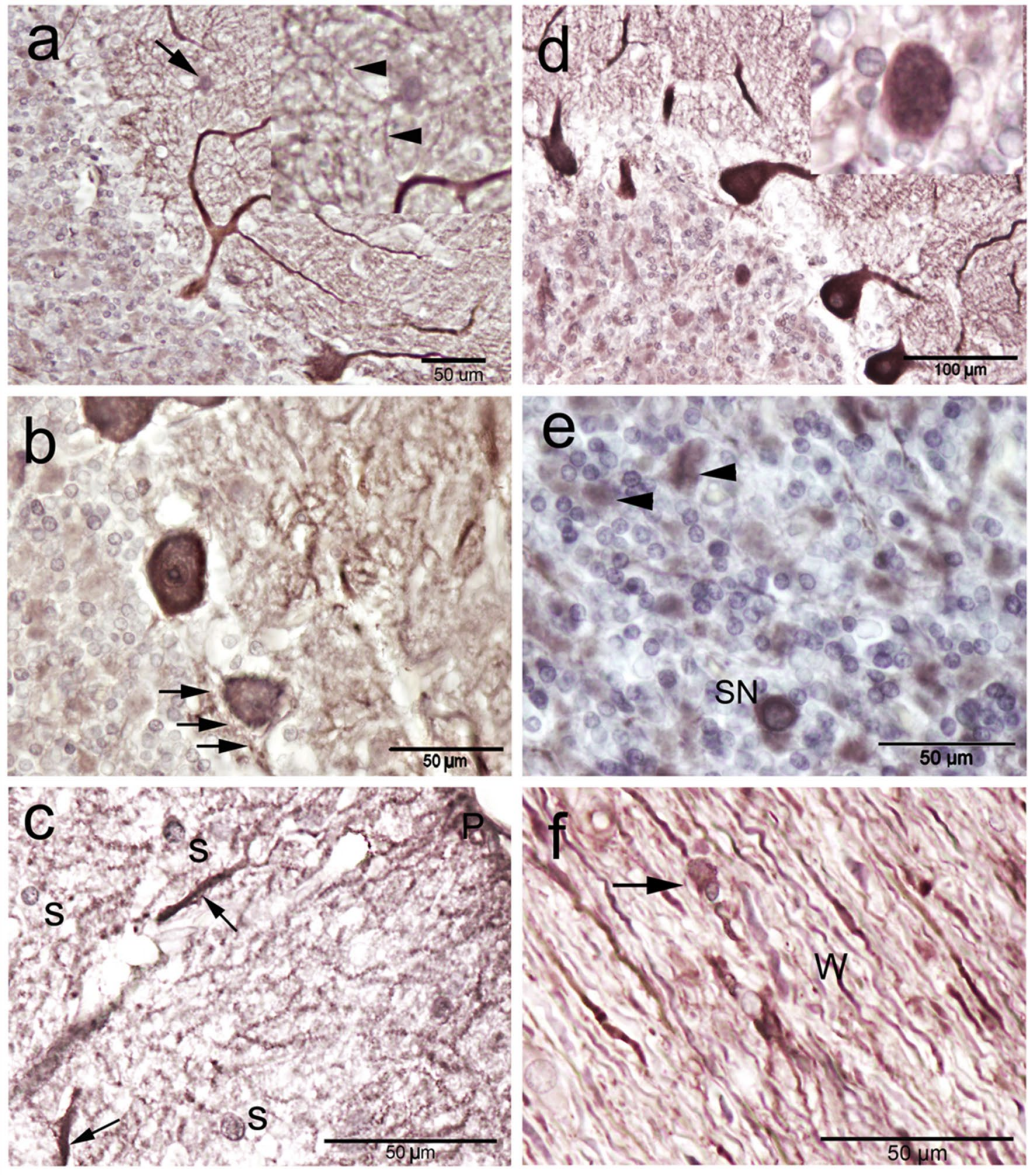
Calbindin-D28k (CB) immunoreactivity in camel cerebellum. (**a**) Basket neuron (arrow) was recognized by their elongated body whose long axis (arrowheads) parallel to the cerebellar surface. (**b**) CB immunopositive rim surrounding the cell body surface of the Purkinje neurons (PC), which corresponding to axon terminals of the basket neurons (arrows). (**c**) The outer zone of the molecular layer showing the polymorphic stellate cells (S). Notice, the strong CB immunoreactivity was also observed within the molecular layer coming from the deeply stained dendrites of PC (arrows) which reaches to the distal ramifications just beneath the pial surface (P). (**d**,** e**) The granular cell layer showing a moderate CB immunoreactivity, however, the expression was heterogeneous. (**d**) An inset showing the Calbindin immunoreactivity by Golgi neurons. (**e**) Synaromatic (SN) large neurons were observed among the granular cell layer. A cellular spots or island in the granular layer (arrowheads) were observed. (**f**) The perivascular large non-traditional neurons (arrowheads) surrounding the blood vessels (BVs) showed positive immunoreactivity with CB in the cerebellar medulla or white matter (W).

In the molecular layer, CB-ir cells showed various degrees of intensity. The immunoreactivity was observed within the cytoplasm of the bodies and extended into the initial segments of the processes. Basket cells were recognized by their shape, orientation and position as well as their elongated somata whose long axes were parallel to the PC layer and localized in the deeper region of the molecular layer (Fig. 6a). Notably, there was CB-ir ring surrounding the somata of the PC, which originated from the axon terminals of the basket cells (Fig. 6b). The stellate neurons were characterized by their rounded/polymorphic somata and their settlement in the outer zone of the molecular layer. The dense CB-ir was also observed within the cytoplasm of PC dendritic tree which extend along the distal ramifications, just underneath the pial surface (Fig. 6c).

In the granular layer, moderate CB-ir was observed in a fraction of the granular cells, however, the expression was heterogeneous. The immunopositive cells displayed moderate, finely granular cytoplasmic immunoreactivity (Fig. 6d,e). The large Golgi neurons showed positive CB-ir (Fig. 6d). There were islands of CB-immunopositive a cellular spots in the granular layer among granule cells (islands of Held) (Fig. 6d,e). The heterogeneous CB expression was also obvious in the medulla, where fibers reacted differently with CB. Moreover, the perivascular large non-traditional neurons showed positive CB-ir (Fig. 6f).

### Parvalbumin (PV) expression in the camel cerebellum

**Figure 7:**
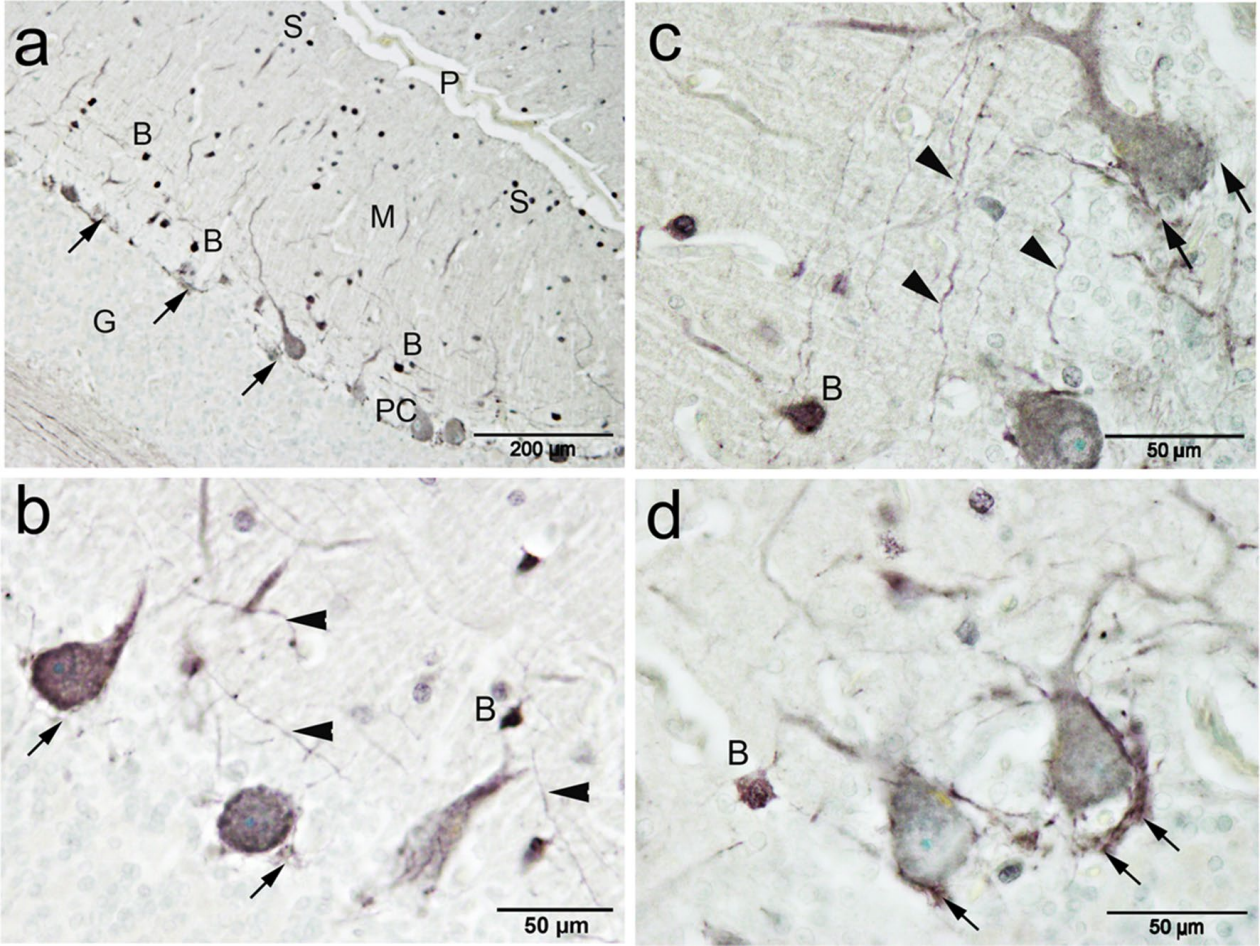
Parvalbumin (PV) immunoreactivity in camel cerebellum. (**a**) Parvalbumin immunoreactivity was observed in the Purkinje cells (PC) and in the stellate (S) and basket cells (B) of the molecular layer cells (m)**. **(**b**) PV was expressed by the Purkinje cells which appeared as a diffuse granular reaction within the cytoplasm of the cell bodies and the dendrites. Notably, the expression was less intense compared to CB**. **(**c**, **d**) The processes of basket cells (B) could be visualized using this marker. The processes (arrowheads) extend from basket cells towards the Purkinje cells and form a network (arrows) surrounding the Purkinje cell bodies. *P* pial surface, *G* granule cells.

**Figure 8:**
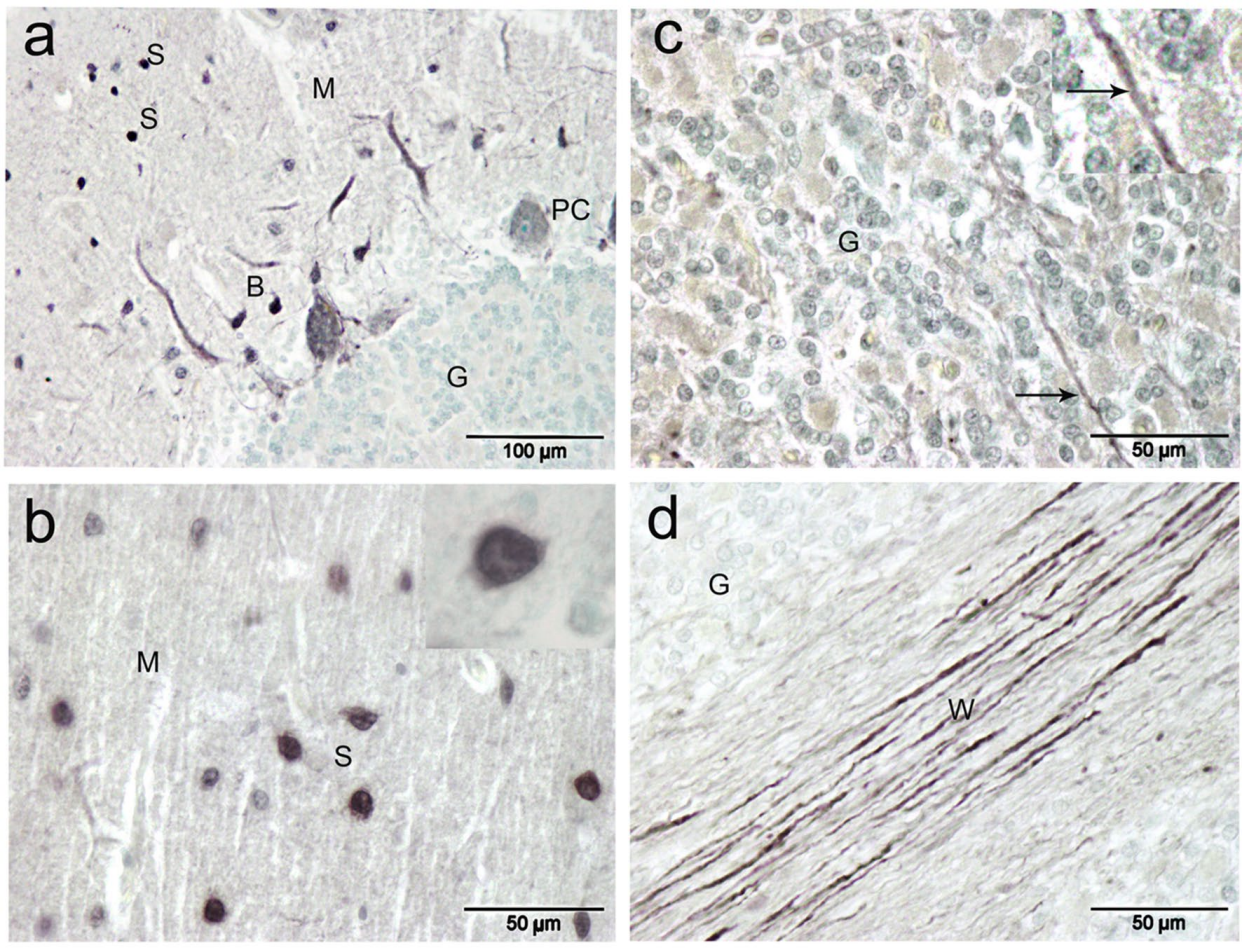
Parvalbumin (PV) immunoreactivity in camel cerebellum. (**a**,** b**) Stellate cells in the upper molecular layer show positive immunoreactivity for PV. (**c**) The axons of Purkinje cells fibers (arrow) traversing the granular layer. (**d**) some fibers of the white matter were expressing PV.

PV-ir was obvious in the PCs and molecular layer cells (Fig. 7a). Parvalbumin was expressed by the PCs which appeared as a diffuse granular reaction within the cytoplasm of the cell bodies and the dendrites, whereas, the nuclei showed less immunoreactivity. Notably, PV-ir was less intense compared to CB (Fig. 7b) and the ratio of PV-immunopositive cells were about 92 percent of the PC. Molecular layer cells showed higher PV-ir compared to CB. Moreover, the molecular layer cells were heterogeneously expressing PV. Its expression was also diffused within the cells with strong expression by the nuclear membrane. The processes of basket cells could be visualized clearly using this marker. These processes extending from basket cells towards the PCs and form a network surrounding the PC bodies (Fig. 7c,d). The stellate cells were expressing PV, with varying degrees of expression in their nuclei. In contrast to PC and basket cells, we could not detect the processes of stellate cells (Fig. 8a,b). We could detect solitary fibers traversing the granular layer, which are likely the axons of PCs (Fig. 8c). Furthermore, some, but not all, fibers of the white matter were expressing PV (Fig. 8d).

### Calretinin (CR) expression in the camel cerebellum

**Figure 9:**
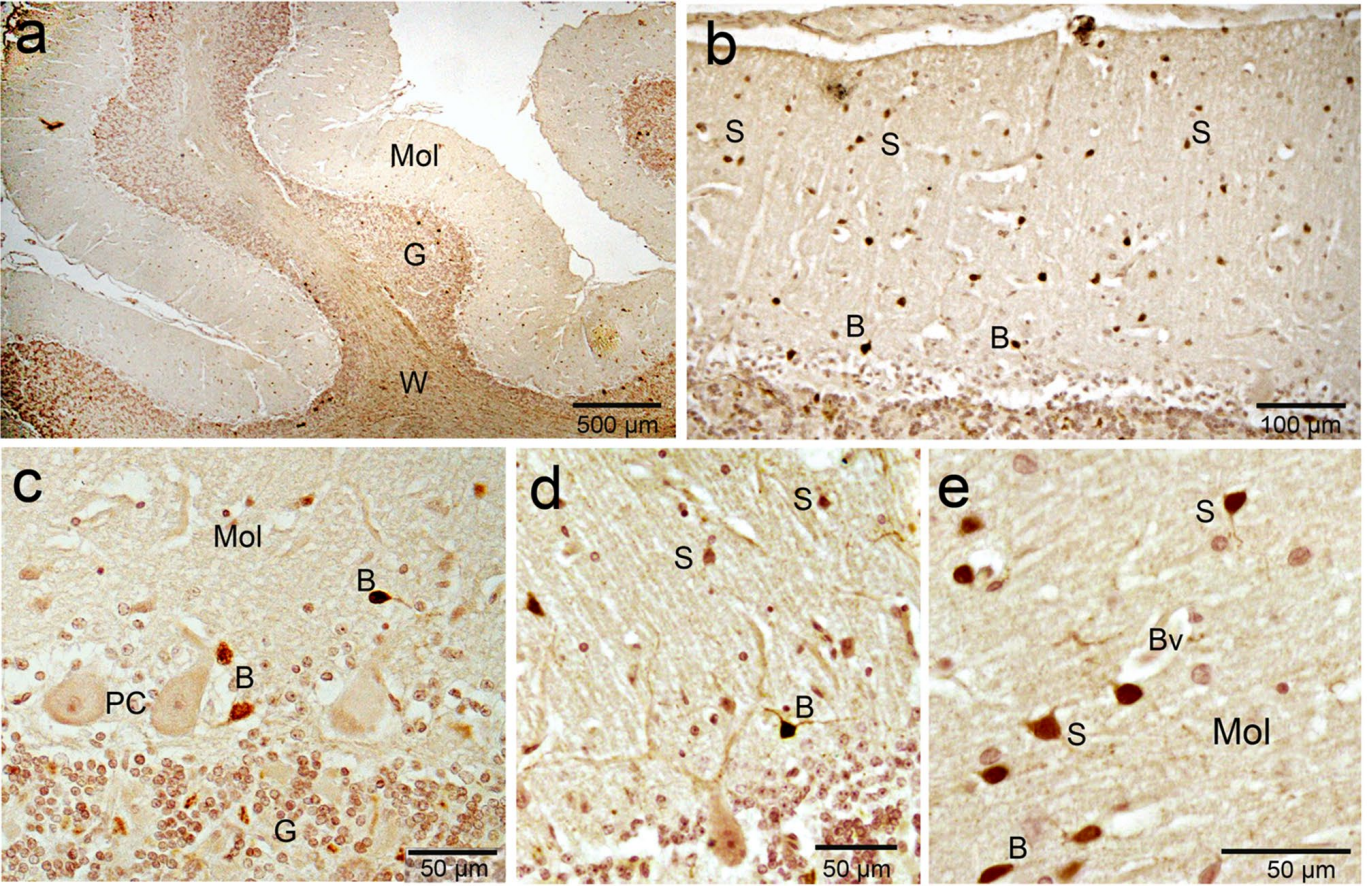
Calretinin (CR) immunoreactivity in camel cerebellum. (**a**) Calretinin immunoreactivity was obvious in almost all cell layers with varying degrees**. **(**b**) The molecular layer cells (M) showed strong immunoreactivity for calretinin. (**c**,** d**) The basket neurons (B) were recognized by their elongated body whose long axis (arrowhead) parallel to the cerebellar surface and localized in the inner region of the molecular layer. Purkinje cells (PC) showed any or very weak immunoreactivity for Calretinin**. **(**e**): The stellate neurons (S) were characterized by their polymorphic body and their localization in the outer zone of the molecular layer**.** *Mol* molecular layer, *BV* blood vessel.

**Figure 10:**
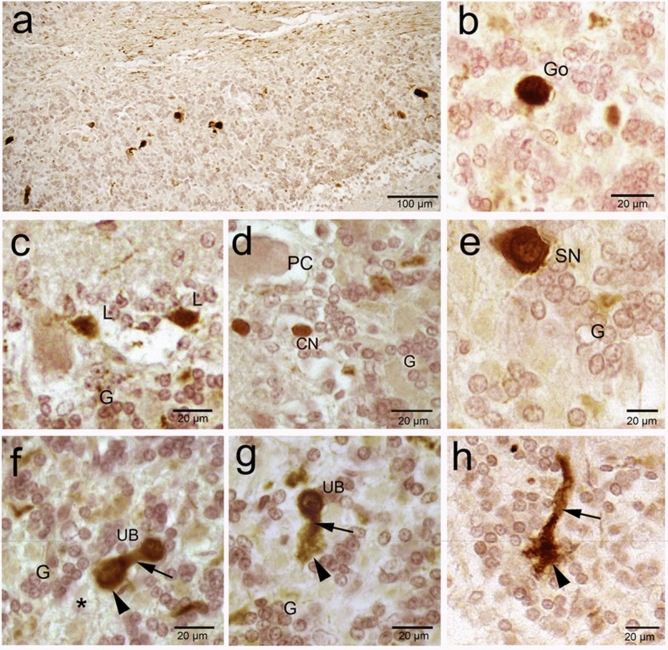
Calretinin (CR) immunoreactivity in camel cerebellum (posterior lobes). (**a**) The granular layer showed moderate positive immunoreactivity for CR in the granule neurons (G) and in the Golgi and other large non-traditional neurons. (**b**) Golgi (Go) neurons showed a voluminous polygonal or ovoid body. (**c**) Neurons of Lugaro (L) showed fusiform and horizontally orientated body. (**d**) Candelabrum neuron (CN) showed pear-shaped body. (**e**) Synaromatic neuron (SN) showed ovoid and horizontally orientated body. (**f**–**h**): Unipolar brush cells (UB) have round/ovoid, vertical cell bodies projecting a single thick process (arrow) that have push-like end (arrowhead).

CR-ir was obvious in almost all cell layers with varying degrees (Fig. 9a). The molecular layer cells showed strong immunoreactivity for CR (Fig. 9a). The density of CR immune positive cells of the molecular layer was 129 cells per 50,000 μm^3^. The basket neurons were recognized by their elongated body whose long axis parallel to the cerebellar surface and localized in the inner region of the molecular layer (Fig. 9 b–d). In addition to the well-known arrangement of the basket cell fibers surrounding the PC somata, we noticed also the dendrites of the basket cells form synapses with dendrites of the Purkinje cells (Fig. 9c,d). The stellate neurons were characterized by their polymorphic body and their localization in the outer zone of the molecular layer (Fig. 9e). PC showed any or very weak CR-ir (Fig. 9c,d) with a ratio of CR-immunopositive cells were about 4 percent of the PC. The granular layer showed positive CR-ir and is composed of two main groups of neurons: the granule neurons (granules) and the large neurons (Fig. 10a). The granule cells have a small, spheroid body and they aggregated in cords or clumps leaving large spaces in between. Large neurons showed stronger CR-ir and have voluminous polygonal or ovoid body. They involve the neuron of Golgi, one of the five traditional corticocerebellar neurons (Fig. 10b). Besides, several other large neuron types, generically indicated as non-traditional neurons were demonstrated. The expression of different CaBP in different cerebellar cell components are summarized in Table 2.

**Table 2:** Intensity of staining in different cerebellar cells.

	CB (fast)	PV (low)	CR (moderate)
Purkinje (Soma)	+ + +	+ +	+
Axon	+ + +	+	−
Dendrites	+ + +	+ +	−
Nucleus	+ + +	+ +	+
Stellate (Soma)	+	+ + +	+ + +
Processes	−	−	+
Basket (Soma)	+	+ + +	+ + +
Processes	+	+ + +	+ + +
Granule (Soma)	+	−	+ +
Golgi (Soma)	+	−	+ + +
Lugaro (Soma)	+ +	−	+
Processes	+ +		+
UBC (Soma)	−	−	+ + +
Processes			+ +
Synaromatic (Soma)	+	−	+ + +
Candelabrum (Soma)	+	−	+ + +

### Non-traditional large neurons

#### Neurons of Lugaro (NL or horizontal neurons)

They are located in the external zone of the granular layer and in more/less close contact with PC layer. Their bodies are fusiform and horizontally oriented, parallel to the surface of the folium. The processes extend from the 2 poles of the body and are oriented horizontally along the boundary between the granular layer and PC and forms synapses with PC. They showed positive immunoreactivity for CB and CR (Fig. 5b and Fig. 10c).

#### Synarmotic neurons (SNs)

They are located in the inner zone of the granular layer near the subcortical white matter. Their bodies are ovoid and horizontally oriented**.** They showed positive reactivity with silver impregnation, CB and CR (Figs. 4f, 6e and 10d).

#### Candelabrum neurons (CN; also known as an intercalated neurons)

These neurons have a vertical major axis and pear-shaped bodies. Their bodies are squeezed against the bodies of Purkinje neurons. These neurons showed positive immunoreactivity for CB and CR (Figs. 5b and 10e).

#### *Unipolar brush neurons (UBN; monodendritic neurons*)

These neurons have round, or ovoid and vertical cell bodies. They are localized throughout the granular layer. A single thick dendrite trunk originates from the external body pole, extends for a short distance and gives rise to a push-like end. These neurons could be visulaized only by its positive CR-ir (Fig. 10 f–h).

## Discussion

The current study aimed to extend the knowledge about the camel cerebellum that helps to learn more about the cerebellum. We demonstrated the detailed characterization of the architecture, both of cellular and fiber components, of the cerebellar cortex in the camel cerebellum. To this end, different histochemical stains were applied. Furthermore, numerous markers were used to visualize the different CaBPs by immunohistochemical analysis. One of the characteristic features in the results of histological stains of the camel cerebellum was that the granular cells were arranged in cords or rosettes^21,22^. According to our observation the cells in the granular layer were not crowded and many spaces were observed.

The relative weight of camel’s cerebellum is 13.86% which is larger than that of donkey 10.8%^23^. When compared to granule cell of other animals, those of camel were the smallest, CSA 35.7 ± 2 µm^2^, where it was 49.7, 50.7, 52.9, 63.8 and 68 µm^2^ in humans, leopards, chimpanzees, tigers and Giraffes, respectively^24^. Granule cell density was 1289 ± 148 cells/0.001 mm^3^, which was more than that of elephants and Bottle-nose dolphin (807 ± 76 and 1083 ± 112 cells/0.001 mm^3^, respectively). It was comparable to that of sheep and common porpoise (1205 ± 131 and 1254 ± 116, respectively). The density was lesser than that of bull, horse and man (1416 ± 141, 1503 ± 165 and 1609 ± 171 cells/0.001 mm^3^, respectively). The density was far lesser than that of rat, mouse and squirrel monkey (3216 ± 287, 3111 ± 341 and 2990 ± 301 cells/0.001 mm^3^, respectively). These results run in parallel to notion that the cell density (Number of cells) decreases with higher brain weight animals^25^.

PC dimension in camel measured 43.76 × 31 µm, with a CSA of 797.73 ± 90 µm^2^. It is larger than that of rat (21 × 25 µm), mice (CSA 188 µm^2^) and cat (width 29 µm), whereas, it is comparable to that of man (30–35 × 50–70) (reviewed in^26^). There three different methods to analyze the PC density; to be PC number with respect to volume in µm^3^, to area in µm^2^ or in certain length (linear). We preferred the later because it is the simplest and not subjected to calculations; however, we presented all three values. Due to the ambiguity inherent in measuring the volume of the PC layer we took the PC density to be number with respect to the area of the PC layer interface rather than to a volume. The units were accordingly neurons/mm^2^^27^. PC linear density was 7.4 ± 1.1 cells/1 mm and the calculated density was 140.8 ± 2 cells/1 mm^2^ or 139.4 ± 12.8 cells/0.001 mm^3^ volume. We thought that the PC densities to be high because of the large body and long extrimites of camel; however, they had the lowest densities among several previously studied species. PC linear density in camel was much less than that of mice (38.5 ± 1.2 cells/mm)^28^. The calculated PC density in camel was also much less than that of rat (930 cells per mm^2^ in the posterior lobe), cat (567 per mm^2^) and monkey (510 cells per mm^2^). PC density in camel was the nearest to that of man (300 cells per mm^2^) among different species; however, it still far less than that of man, about half the densities (reviewed in^26^). These low PC and granule cell densities were recorded also in the African elephant^29^. Together with the abundant cerebellar islands, this might indicate an abundant synapses in the neuropil to control the complex motor patterns and to process the sophisticated neural information which increases during phylogenesis^29^.

The CaBPs are widely expressed throughout the nervous system however they are among the most abundant proteins in the cerebellum^8^. These proteins expressed in the peripheral tissues including the expression of CR in the thymus, CB in the kidney and PV in the skeletal muscles and endocrine tissues^15,30^. In fact, no data on the expression of the CaBP within the camel cerebellum are available to date. Therefore, the results of the current study provide for the first time the detailed characterization of cellular, together with fiber, components in the camel cerebellar cortex. To this end, different immunohistochemical analyses were applied using markers for CB, CR and PV. We observed that the expression of each molecule was differentially expressed among different cerebellar populations.

As a ubiquitous second messenger, Ca^2+^ has been shown to regulate membrane excitability^31^, dendrite development^32^, synaptogenesis^33^, and many other processes contributing to the neuronal primary functions of the information processing and memory storage^34^.

CB was detected in the nervous system^35^, after its discovery in the chicken intestine^36^. Its expression is started early in cerebellar development in fetuses and newborn kittens during neuronal migration and is affected in aged dogs^37^. CB expression was intensely and widely distributed in different compartments; neuronal bodies, their main processes and the axon and dendritic terminal compartments, thus indicating its importance for normal cerebellar function. This some studies of various mammalian species stated that CB expression was predominant in Purkinje neurons^38,39^, the present study demonstrated that the CB was expressed in almost all cortical neuron types, however, with different intensity. The PCs showed the highest CB immunoreactivity, which distributed throughout the cytoplasm of cell bodies, axons and dendritic tree. Therefore, CB demarcates the characteristic morphology of the Purkinje neurons throughout its extent. However, a few low CB-immunopositive PC bodies were observed. The appearance of the detailed morphology of PC, including its very distal tiny branchlets and spines, using these antibodies, made it a marker of choice to study PCs^40^. The cytoplasmic abundance of CB is consistent with the previous studies that demonstrated the interaction between CB and cytoplasm proteins, such as myoinositol mono-phosphatase and protein M^41^. Additionally, the nuclear localization of CB in the PC supports the hypothesis that is plays a role in the regulation of gene expressions^42^. This homogeneous, intense expression of CB along the entire cytoplasm of the PC indicates the involvement of CB in many neuronal functions, especially in this large neurons which receive huge numbers of synapses from numerous types of cells. Furthermore, the experimental data demonstrated that CB plays a neuroprotective role against the oxidative stress or toxic effect of prolonged stimulation by excitatory amino acids (i.e.: glutamate; aspartate)^43^. Therefore, the absence or alteration in the expression of calcium-buffer proteins by any way will result in marked abnormalities in cell firing, with alterations in simple and complex spikes^37^. This will be accompanied by many neurological disorders and neurodegenerative conditions concerning motor coordination and sensory functions^44^. It has been reported that PC loss CB-ir in some viral encephalitides from HIV encephalitis in humans^45^, rabies-infected cattle^37^, neonatal Borna disease infection in rats^46^, and experimental infection of in mice^47^, or aging in dogs^48^. These notions postulate a relationship between loss of CB-ir in PC, altered calcium homeostasis and calcium internal buffering and dysfunctions in GABAergic neurotransmission. The current study supplies surprising findings of the expression of CB within different neuronal populations such as the non-traditional large neurons (lugaro and synaromatic neurons) and granule cells in the granular layer and other inhibitory GABAergic neurons (basket and stellate cells) in the molecular layer of the camel cerebellar cortex. Our results are in line with that observed the positive immunoreactivity for CB-D28k in the bovine GABAergic inhibitory interneurons such as the basket cells, Golgi and Lugaro neurons , elephant^25^ sheep^49^**.** The positive immunoreactivity for CB was also detected within the stellate and basket cells of the human cerebellar cortex in the molecular layer. Recently, the immunoreactivity was observed in the granule neurons, and in the non-traditional large neurons such as the candelabrum, ellpoisidal, Lugaro, and the synaromotic neurons^42^. Therefore, we concluded that CB in the camel cerebellum plays a primary role in the regulation of cerebellar cortex functions, in addition to, its main role in integrative functions of the cerebellar cortex, which involoves sensorimotor and cognitive functions^42^. CB Purkinje neurons are known as the main source of projective axons and the sole output from the cerebellar cortex. SNs have recently been reported as the second type of projective neurons in the cerebellar cortex which showed positive CB-ir^50^. The expression of CB was linked to the GABAergic projective neurons (Purkinje and SN), and we assumed that CB is mainly associated with the neurotransmission of inhibitory type^42^. In line with this notion, the current study demonstrated the expression of CB in the GABAergic inhibitory neurons including stellate, basket, and Lugaro neurons^51,52^.On the other hand, we found also that CB expression was also demonstrated in some glutamatergic excitatory neuron including a large subpopulation of the granule neurons. Taken together, our results indicate the expression of CB by neurons is implicated in both the projective (extrinsic) and regulatory (intrinsic) circuits of the camel cerebellar cortex and there is no correlation between CB expression and type of neurotransmitter^42,52^.

PV was expressed by PC, however, its intensity in the soma and dendrites was lower when compared to CB. Moreover, PV showed variations in the staining intensity within the PC cells. The same results were demonstrated in the cerebellum of rat, primates and human^8,35^. Two subpopulations within the molecular layer were also expressing PV, the stellate and basket cells^53^. These inhibitory GABAergic interneurons receive excitatory inputs from the granule cells and climbing fibers and exert inhibitory signals to the PC^54^. Both populations have relatively comparable morphologies with short dendritic trees, which are organized in an almost right angle to the dendrites of the PC cells. The axons of basket cells extend and, as their name implies, organized around the PC somata as a basket^47^ The dendrites of both cells and the axons of the stellate cells form synapses on the dendritic tree of Purkinje cells. In contrast to the human cerebellum, we found that PV was not expressed by the GABAergic Golgi cells within the granular layer of the camel cerebellum^54^. Although the PCs have both CB and PV, the autism spectrum disorder (ASD) was linked to PV reduction. The cerebellum neuropathology in ASD was first detected over 35 years ago^55^, where the significant reduction in PV-expressing PC in autistic individuals is considered a histopathological feature of autism^56^. Therefore, altered calcium metabolism plays a key role in ASD pathophysiology, where it might, in turn, impact GABAergic signaling^57^. PV expressing neurons are more subjected to degeneration. In contrast, CR and/or CB expressing neurons are more likely resistant reviewed in^58^. CR is structurally related to CB and was first described in 1987 in embryonic chick retina, hence its name is driven from CB and retina^59,60^**.** According to the speed of Ca^2+^ buffering, CB is the fastest, PV is the slowest and CR is sharing some kinetic properties of both fast and slow buffers in modifying Ca^2+^ transients due to the cooperative Ca^2+^ binding of CR^59–61^. In the camel cerebellum, the CR is expressed in almost all cells except the PC which showed any or very weak CR-ir. However, recently, it has been reported that there is CR-ir in the migrating PC from the ventricular neuroepithelium in the developing human fetal cerebellum^62^, which suggest a possible role during migration. This observation was in line with Álvarez et al., 2008 who demonstrated the variability for calretinin expression in the purkinje cells of the sheep. The authors claimed the great variability in CR-immunopositivity cannot be associated to methodological variables instead, this notion confirms the idea of a heterogeneous functional organization for the cerebellar cortex within an apparent homogeneous anatomical and histological structure. In camels, The majority of granule cells were expressing CR, however, was relatively weaker than other cells. This was in line with that reported in rodents^63–65^. Moreover, CR-ir was detected also in the axons of granule cells, the parallel fibers, suggesting an important role in Ca^+2^-dependent synaptic plasticity^65,66^.

CR was also expressed by large non-traditional neurons of the granular layer: unipolar brush cells (UBC), Lugaro cells, synaromatic cells and candelabrum cells. These populations were more intensely stained with CR antibody than the surrounding granule cells, supporting the notion that they contain higher concentrations of CR. Schwaller, et al.^54^ and colleagues reported only that CR is expressed by unipolar brush cells (UBC)^67^ and Lugaro cells in rodents^68^. The authors did not mention whether other large non-traditional neurons expressed CR**.**^68^. Lugaro cells and their processes are usually located just below the PC layer, which we could detect CB and even using the Luxol fast blue staining. They are believed to exert an indirect inhibitory influence on PC via a glycinergic inhibition of Golgi cells^69^. CR-ir by Lugaro cells is not conserved in all vertebrates, because Lugaro cells of Pigeon are not expressing it, instead they express secretagogin^70^. We found UBC in the granule cell layer of the posterior cerebellar lobe in camel, similar to that described in rodents^65^ and sheep^71^. They have a single, thick process which forms a bush-like tip which is reported to function as excitatory glutamatergic neurons^67^. We found distinct large CR-ir globular cells deep in the granular layers suggestive to be Golgi cells. However, it has been reported that Golgi cells in all species except for guinea pig are immunonegative for calretinin^72^. It has been suggested recently that these cells might be a subtype of Lugaro cells in pigeon, where there are both globular and fusiform Lugaro cells^70^. These results might be indicate that CR expression is not conserved in certain cells among all vertebrates.

The neuroprotective role of CR against calcium-stimulated cytotoxicity has been suggested^60^. Because of the crucial role played by Ca^+2^ in neuronal physiology, it is not surprising that even modest impairments of Ca^+2^ homeostasis result in profound functional alterations. Despite the heterogeneous etiology of neurodegenerative disorders, as well as the physiological aging process, are all characterized by disruption of Ca^+2^ homeostasis and signaling.

## Conclusion

In summary, the results of the current study achieved a complete map for the neurochemical organization of CaBP expression and distribution by different populations in the camel cerebellum. Understanding of these data under normal conditions represents a prerequisite for studying the cerebellar pathophysiology. PCs are expressing high CB, low CR and moderate PV. Molecular layer cells share the same expression profile; low CB and high CR and PV. Granule cells are expressing heterogeneous (low/moderate) CB, moderate CR and no PV, whereas this phenotype is the reverse in Lugaro cells. Golgi, UBC and synaromatic share the high CR-ir and no PV-ir and low CB-ir, however, UBC were not expressing CB.

## Supplementary information

Supplementary Informaion.
