# Supplementary material for: An updated investigation on the dromedary camel cerebellum (Camelus dromedarius) with special insight into the distribution of calcium-binding proteins
Source: Sci Rep. 2020 Dec 3;10:21157. doi: 10.1038/s41598-020-78192-7 (PMC7713137; doi:10.1038/s41598-020-78192-7)
Supplement: Supplementary file 1 — Supplementary Informaion. [file 41598_2020_78192_MOESM1_ESM.docx]

**An updated investigation on the dromedary camel cerebellum *(Camelus dromedarius)* with special insight into the distribution of calcium-binding proteins**

**Abdelraheim H. Attaai^1^, Ahmed E. Noreldin^2^, Fatma M. Abdel-maksoud^1^, Manal T. Hussein^1^**

^1^Department of Anatomy and Histology, Faculty of Vet. Medicine, Assiut University, 71526, Egypt

^2^Department of Histology and Cytology, Faculty of Veterinary Medicine, Damanhour University, Damanhour 22511, Egypt

Manal T. Hussein and Abdelraheim H. Attaai are contributed equally to this work

**Corresponding author:**

Dr. **Fatma M. Abdel-maksoud**

Lecturer of anatomy and histology

Department of Anatomy and Histology, Faculty of Veterinary Medicine, Assiut University, 71526, Assiut, Egypt

[Fatma.abdelmaksoud@vet.au.edu.eg](mailto:Fatma.abdelmaksoud@vet.au.edu.eg)


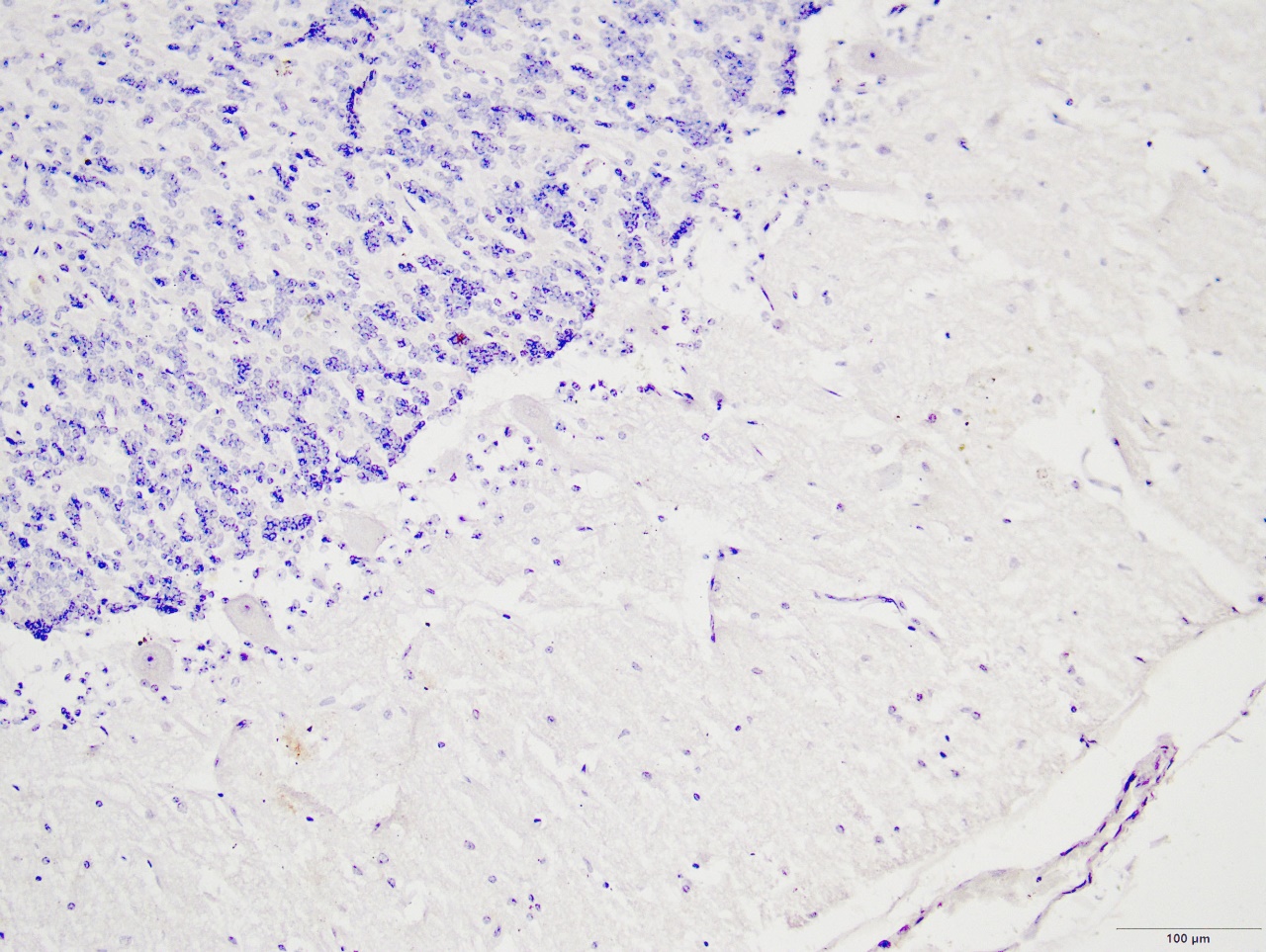


Figure suppl 1: Negative control was performed without incubation with any primary antibody. No staining indicates the specificity of used primary antibodies. The section counterstained with HX.


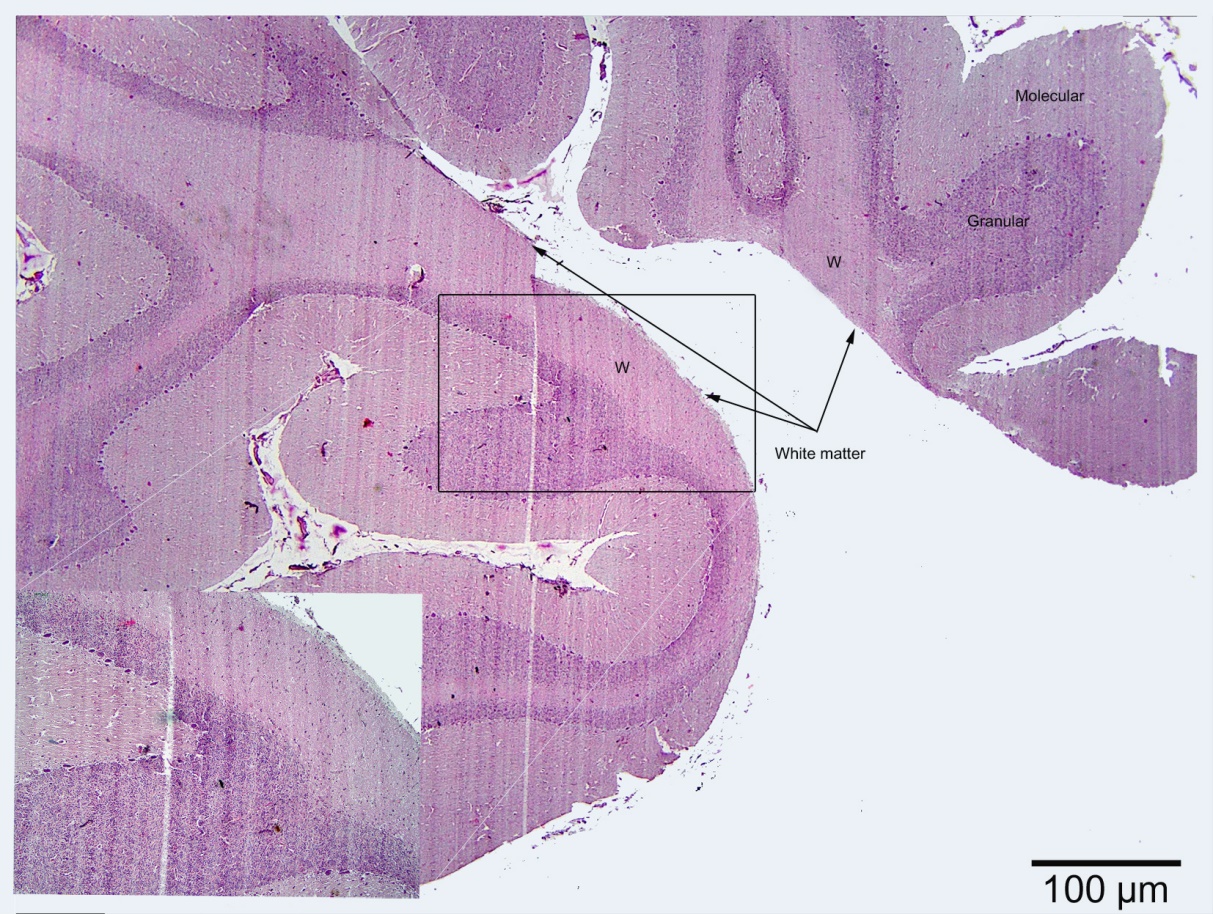


Figure suppl 2: A photograph showing a wide region of white matter which reaches distally to the pial surface, without covering with cortical tissue (arrows). Hx&E stained section


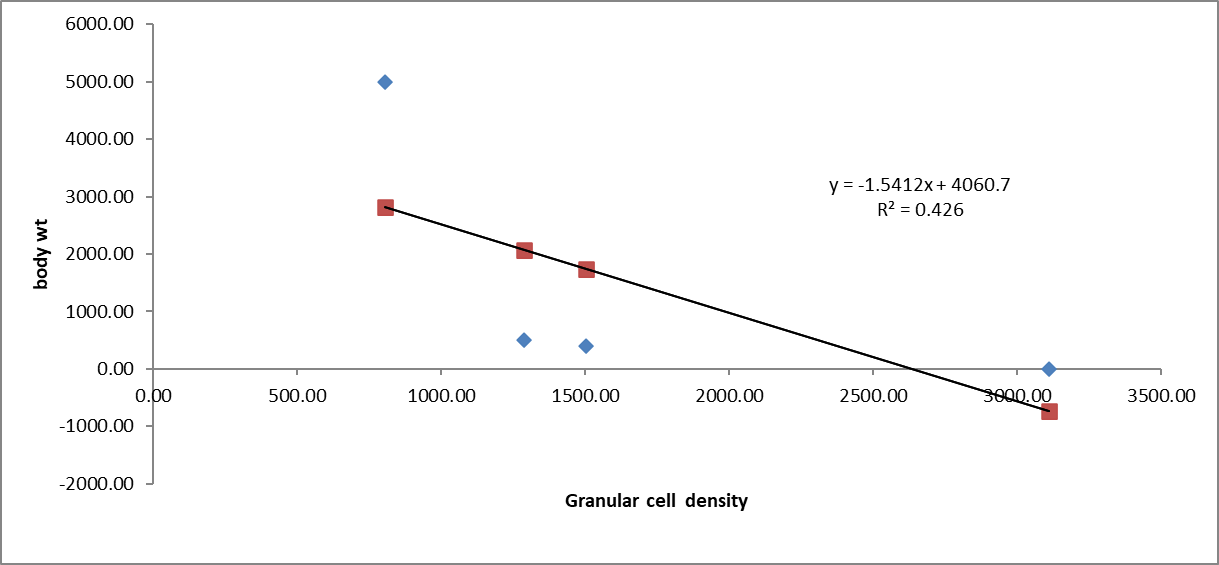


Figure suppl 3 showing the regression of granular cell density on the body weight of 4 different animals (the elephant, horse and mouse obtained from Lang et al 1975) and our data of camel. The calculated correlation, (the negative value -0.65), means that the granular cell density decreases when the body weight increase. The data were analyzed using data analysis tool of Microsoft Excel software.


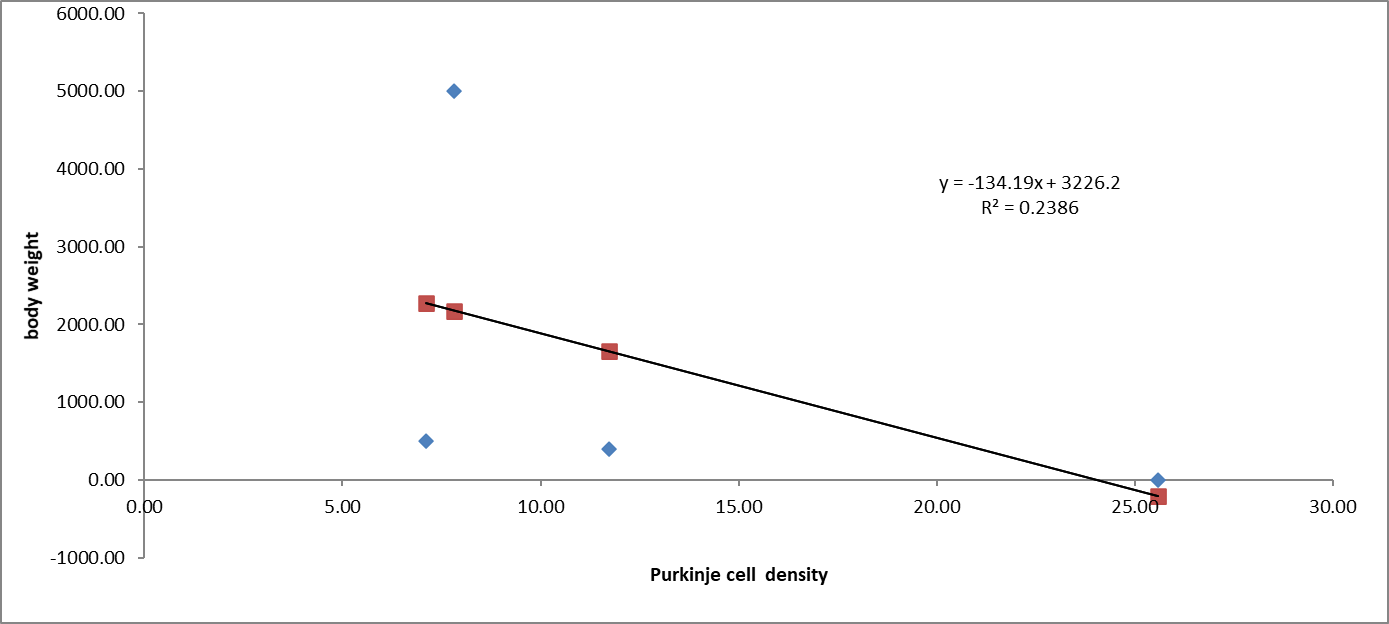


Figure suppl 4 showing the regression of Purkinje cells density on the body weight of 4 different animals (the elephant, horse and mouse obtained from Lang et al 1975) and our data of camel. The calculated correlation, (the negative value -0.49), means that the PC density decreases when the body weight increases. The data were analyzed using data analysis tool of Microsoft Excel software.
